# Human Anti-Oxidation Protein A1M—A Potential Kidney Protection Agent in Peptide Receptor Radionuclide Therapy

**DOI:** 10.3390/ijms161226234

**Published:** 2015-12-18

**Authors:** Jonas Ahlstedt, Thuy A. Tran, Sven-Erik Strand, Magnus Gram, Bo Åkerström

**Affiliations:** 1 Section for Infection Medicine, Department of Clinical Sciences in Lund, Lund University, Lund 221 84, Sweden; magnus.gram@med.lu.se (M.G.); bo.akerstrom@med.lu.se (B.A.); 2 Lund University Bioimaging Center, Lund University, Lund 221 84, Sweden; thuy.tran@med.lu.se; 3 Section of Medical Radiation Physics, Department of Clinical Sciences in Lund, Lund University, Lund 221 84, Sweden; sven-erik.strand@med.lu.se

**Keywords:** A1M, radioprotection, octreotide, PRRT, oxidative stress, antioxidation, kidney, glomerulus, tubule

## Abstract

Peptide receptor radionuclide therapy (PRRT) has been in clinical use for 15 years to treat metastatic neuroendocrine tumors. PRRT is limited by reabsorption and retention of the administered radiolabeled somatostatin analogues in the proximal tubule. Consequently, it is essential to develop and employ methods to protect the kidneys during PRRT. Today, infusion of positively charged amino acids is the standard method of kidney protection. Other methods, such as administration of amifostine, are still under evaluation and show promising results. α_1_-microglobulin (A1M) is a reductase and radical scavenging protein ubiquitously present in plasma and extravascular tissue. Human A1M has antioxidation properties and has been shown to prevent radiation-induced *in vitro* cell damage and protect non-irradiated surrounding cells. It has recently been shown in mice that exogenously infused A1M and the somatostatin analogue octreotide are co-localized in proximal tubules of the kidney after intravenous infusion. In this review we describe the current situation of kidney protection during PRRT, discuss the necessity and implications of more precise dosimetry and present A1M as a new, potential candidate for renal protection during PRRT and related targeted radionuclide therapies.

## 1. Peptide Receptor Radionuclide Therapy (PRRT)

### 1.1. Background

Neuroendocrine tumors (NETs) are a large group of neoplasms derived from the neuroendocrine system. Common sites for primary tumors are the gastrointestinal tract, the lungs, and other neuroendocrine tissues [1,2]. In the 1980s it was discovered that some tumors overexpress peptide receptors. The somatostatin receptor family consists of five receptor subtypes, sstr1-5 [3]. A majority of NETs has a strong over-expression of somatostatin receptors, mainly subtype 2 (sstr2), which is the key target for therapy using stable somatostatin analogues (SSA) [3]. Peptide receptor radionuclide therapy (PRRT) using somatostatin analogues such as octreotide chelated to β-emitting radionuclides (^177^Lu, ^90^Y) have been successfully used to target inoperable or metastatic NETs for the past 15 years. A large body of evidence describes the effectiveness and clinical safety of these procedures [3]. PRRT as a therapeutic option, has proven ideal for patients expressing well or moderately differentiated neuroendocrine carcinomas of grade 1 or 2, since these tumors often have metastasized and are inoperable. The first attempt to evaluate the diagnostic compound ^111^In-octreotide as a potential radiopharmaceutical took place in the 1990s, with patients receiving cumulative activities of at least 20 and up to 160 gigabecquerel (GBq; activity unit for radioactivity 1 Bq equal to one decay per second). Therapeutic effects could be demonstrated: partial and minor remission and stabilization of progressive tumors in half of the patients [4]. Side effects consisted of mild bone marrow toxicity and leukemia with absorbed doses to the bone marrow in the order of 3 Gray (Gy). However, the failure of the therapy in many patients can be explained by the short range of the emitted electrons (mainly Auger electrons) in combination with heterogeneous receptor expression (equal to activity distribution) in the NETs. Because of this, ^111^In-based therapy is generally not recommended for PRRT [5]. Although ^111^In-octreotide infusion to this day remains a therapeutic option for NETs, it has been replaced by conjugates with β-emitters such as ^90^Y and ^177^Lu.

PRRT utilizing the two radiopharmaceuticals ^90^Y-[DOTA^0^, Tyr^3^]-octreotide (DOTATOC) and ^177^Lu-[DOTA^0^, Tyr^3^]-octreotate (DOTATATE) has proven to be an effective and safe alternative treatment for patients with metastatic NETs [6,7]. Both peptides are derivatized somatostatin analogues that show high affinity for sstr2. DOTATATE has a higher affinity for sstr2 (1.5 ± 0.4 nM) when compared to DOTATOC (14 ± 2.6 nM), but does not display any affinity for other subtype receptors such as sstr3 and sstr5 [8]. DOTATOC has a weak, but non-negligible affinity, for sstr3 and sstr5 (393 ± 84 nM and 880 ± 324 nM respectively) [9].

DOTATATE has been shown to perform favorably compared to alternative treatment modalities in terms of tumor response rate and progression-free survival with a survival benefit of 40–72 months when compared to controls [10]. A recent large international multi-center trial [11] has shown marked improvement in progression-free survival rate and overall survival in patients undergoing PRRT with DOTATATE.

### 1.2. Diagnostic Assessment

The WHO recently published a three-tiered classification system for NETs based on their mitotic rate and Ki-67 index: NET Grade 1, NET Grade 2, and neuroendocrine carcinoma [12]. Patients with NET grade 1 or 2 (low grade carcinomas) are ideal candidates for PRRT. The sstr2-expressing NETs in the bronchial and gastroenteropancreatic tract are the most common NETs considered for PRRT with radiolabeled somatostatin analogues [3]. Other NETs, such as medullary thyroid carcinoma, phaeochromocytoma, paraganglioma and neuroblastoma, are also highly suitable candidates for PRRT [13,14,15,16,17,18].

In order to select viable candidates that would benefit from PRRT it is important to assess sstr-status. Functional imaging with single photon emission computed tomography (SPECT) using ^111^In-pentetreotide and positron emission tomography (PET) with ^68^Ga-labaled SSA are well established methods to assess sstr status and collect essential information for staging and re-staging [19,20]. In particular, PET has proven superior to SPECT, providing a more accurate, personalized treatment [21]. A detailed anatomical imaging of the primary tumor and its metastases can be obtained utilizing computed tomography (CT), ultrasonography and magnetic resonance imaging (MRI) and is important to evaluate treatment response [3].

Contraindications for PRRT treatment include pregnancy, acute concomitant diseases, and psychiatric disorders [3]. Other considerations are compromised bone marrow or severely compromised renal function, although patients with the latter can still be considered for treatment.

### 1.3. Dose-Limiting Organs

The bone marrow and the kidneys are both dose-limiting organs in PRRT-therapy, and the latter is considered to be the major dose-limiting organ [22,23]. As there is physiological renal retention of radiolabeled somatostatin analogues, the cumulative kidney absorbed dose limits the possible activity to administer. If one can reduce the toxicity to normal tissues (mainly the kidneys), the therapeutic window can be enlarged. Radiolabeled somatostatin analogues are filtered by the glomeruli and reabsorbed in the proximal tubules. The megalin/cubulin system plays an essential role in the reabsorption of octreotide. Reduction of the renal uptake can be achieved during PRRT by infusion of the positively charged amino acids l-lysine and l-arginine [3].

## 2. Dosimetry

Because of the unwanted uptake in the kidneys and other non-target tissues, precise and reliable dosimetry is needed to optimize PRRT. Absorbed dose is defined as imparted energy per mass unit (1 J/kg equals 1 Gy). The imparted energy causes ionizations and excitations inducing damage to biological molecules due to direct or indirect effects. The latter process include reactions with free radicals that are generated as a result of interaction with water molecules. Examples of direct and indirect effects of the deposited energy in biological tissue are oxidations and radical formations of cellular molecules, causing single strand (SSB) and double strand breaks (DSB) of nuclear DNA.

*In vivo* dosimetry requires knowledge of the kinetics of the radiopharmaceutical. However, the determination accuracy of the radioactivity distribution in imaging techniques such as SPECT and PET becomes limited. For example, the quantitative uncertainty is 2% or more using SPECT [24].

In conventional external radiotherapy, an absorbed dose of 23 Gy to the whole kidney gives an expected risk of 5% of nephrotoxicity within five years [25]. This cannot be applied directly to PRRT due to the lower dose rate where absorbed doses of 27–29 Gy are tolerated. For the bone marrow a maximal absorbed dose of 2 Gy is generally acceptable. This emphasizes the importance of individualized kidney and bone marrow dosimetry, in order to predict and circumvent toxicity and maximize the absorbed dose to the tumor. A threshold biological effective dose (BED) to the kidneys for patients with no risk factors has been evaluated by Bodei *et al.* [22] to 40 Gy. While a maximum BED of 28 Gy to the kidneys is recommended, the same author states that further investigation is needed to determine a more definite threshold BED.

The varying activity distributions within the kidney affect the absorbed dose distribution. High-energy β emission from ^90^Y results in a fairly homogeneous absorbed dose distribution, whereas low-energy electron (Auger electron) and medium energy β emitters as ^111^In and ^177^Lu give more inhomogeneous absorbed dose distributions. As a consequence in PRRT, a higher absorbed dose can be seen in cortical nephrons and in the juxtamedullary zone.

Guidelines on how individualized patient dosimetry in radionuclide therapy can be performed are found in the literature [26,27]. The relative activity distribution *in vivo* is quantifiable by SPECT and PET. With a pure, low energy β emitter this activity concentration can simply be transformed to an absorbed dose rate by multiplying with an absorbed dose conversion factor. If a substantial fraction of the decay energy is released as photons or high energy β particles, radiation cross-fire has to be considered.

In most cases dosimetry is performed using the Medical Internal Radiation Dose (MIRD)-concept calculating mean absorbed dose D, with standardized human anatomy. The MIRD scheme is in a simplified way written as

(1) $$D=Ã\times S=A_{0}\times \tau \times S$$

where Ã is the cumulated activity and *S* is the mean absorbed dose per cumulated activity (or *S*-factor), which depends on the properties of target organs and the radionuclide used. Several numerical or compartment models are used to calculate Ã and dedicated software such as OLINDA is then used to calculate the absorbed dose [28,29]. Patients with known risk factors can benefit from patient specific dosimetry, where the cumulated dose is determined from a series of scintillation camera measurements using either planar scintigraphy or SPECT. Reported absorbed dose range per unit activity to the kidneys for ^90^Y-DOTATOC and ^177^Lu-DOTATATE treatments are 2.55–2.84 Gy/GBq and 0.62–0.9 Gy/GBq, respectively (Table 1) [3].

**Table 1:** Three examples of reported absorbed doses in kidneys per unit activity (Gy/GBq) with mean ± SD or median range (marked with an asterisk) following PRRT utilizing ^90^Y-DOTATOC and ^177^Lu-DOTATATE.

	^90^Y-DOTATOC (Gy/GBq)	^177^Lu-DOTATATE (Gy/GBq)
Absorbed dose per unit administered activity	2.84 ± 0.64	0.88 ± 0.19
	2.44 (1.12–4.5) *	0.62 (0.45–17.74) *
	2.73 ± 1.41	0.9 ± 0.3

* median (range).

Based on the kidney anatomy, a detailed dosimetry model has been developed by the Committee on Medical Internal Radiation Dose (MIRD). Here, regional *S*-factors can be derived and used in the case of heterogeneous activity distribution [30].

It has been reported that high energy β emitters as ^90^Y can be accurately described by the MIRD kidney dosimetry approach whereas low energy emitters as ^111^In and ^177^Lu need dosimetry calculations based on autoradiography activity distributions [25].

### 2.1. PRRT—Side Effects and Protective Measures

Acute side effects, such as headaches, nausea, and vomiting occur in the majority of patients [31]. These side effects can be ameliorated with hydration and antiemetic treatment. Delayed side effects are closely related to the dose limiting organs and include loss of renal function, especially in patients with severe hypertension, diabetes mellitus, and bone marrow toxicity [18].

Most of the radiolabeled somatostatin analogs are cleared by the kidneys and the majority of the activity is excreted via the urine. However, a small amount (about 2%) is reabsorbed and retained in the proximal tubuli and prolonged irradiation of the kidneys follows. To inhibit the uptake in the proximal tubule protective measures are taken by infusing positively charged amino acids such as l-lysine and/or l-arginine. These countermeasures have proven to significantly reduce the renal absorbed dose and are widely used today.

Despite protective measurements, loss of renal function has been observed in patients undergoing PRRT. It has been observed that patients with NETs treated with ^177^Lu-DOTATATE or ^90^Y-DOTATOC suffered a creatinine clearance loss of 3.8% and 7.3%, respectively, per year [3]. The infusion of basic amino acids has also been reported to potentially cause acute hyperkalemia as well as being a potential catalyst for hormonal crisis in patients undergoing PRRT [32,33].

The search for methods that could reduce renal uptake and mitigate radiation induced damage requires extensive knowledge of uptake mechanisms, kinetic properties of the protective agents, and their distribution in target organs and other tissues.

The cytoprotective agent amifostine has demonstrated protective properties. In vivo experiments with rats showed a significant decrease in renal uptake [34]. To further increase protection, the combined infusion of amifostine and l-lysine has been suggested in order to cover both long and short-term effects.

### 2.2. Kidney Activity Distributions

Autoradiography of resected kidneys from patients administered ^111^In-DTPA-Octreotide has revealed irregular distribution of activity in the cortex with highest uptake around the juxtamedullary cortex region [25]. It was also reported that the medulla showed heterogeneous activity distribution with highest activities in the central parts.

**Figure 1:**
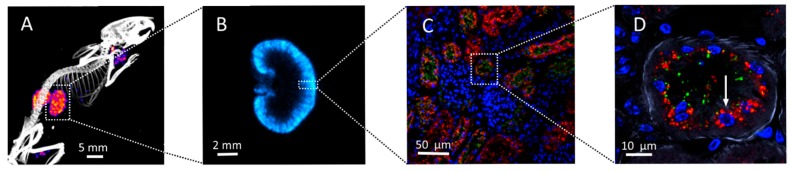
Visualizing the distribution of ^125^I-labelled A1M 20 minutes post-injection at different magnification using SPECT/CT (**A**) and digital autoradiography (**B**) at the organ-level and fluorescence imaging showing both A1M (**green**), octreotide (**red**) and cell nuclei (**blue**) (**C**,**D**) at cellular and sub-cellular levels. The arrow in (**D**) indicates one single cell. The figure is modified from reference 35 with permission from the publisher.

To fully understand the biological effect of the heterogeneous activity distribution and corresponding absorbed dose distribution, the cellular and subcellular pattern need to be revealed. In the study from Ahlstedt *et al.* [35] it was shown that a fluorescence-labeled octreotide-derivate was localized preferentially to the kidney cortex in tubular epithelial cells (See Figure 1 for more details).

Assuming a mean absorbed dose to the cortex of 27 Gy, local maximum absorbed doses for ^177^Lu, ^111^In, and ^90^Y have been reported to be 47, 40, and 25 Gy respectively [25]. Furthermore, the JM complex has been reported to receive absorbed doses of 39 Gy for ^111^In-DTPA-octreotide and 32 Gy for DOTATOC with an average kidney absorbed dose of 27 Gy. For DOTATATE an average kidney absorbed dose of 23 Gy can result in an absorbed dose of 40 Gy to the JM complex [25].

## 3. Oxidative Stress

### 3.1. Oxidation and Antioxidation

The term “oxidative stress” is used to describe physiological conditions with an abnormally high production of redox active compounds and/or impaired antioxidative tissue defense systems [36]. Mediators of oxidative stress are reactive oxygen species (ROS) including free radicals, which are highly reactive due to the presence of unpaired electrons. ROS and radicals react with proteins, DNA, membranes etc, which can lead to unwanted modifications of the target molecules and loss of their functions. Endogenous generators of oxidative stress are hemoglobin (Hb) and heme, released during hemolysis [37] and other heme-proteins such as cytochrome c and myoglobin, released during inflammation and tissue necrosis. Mitochondrial respiration also generates ROS, and is a source of oxidative stress during inflammation and tissue necrosis.

Physiological defense systems have evolved to counteract the chemical threat of oxidative stress to exposed tissues and macromolecules. ROS and other oxidative compounds are inhibited and eliminated by antioxidation enzymes such as superoxide dismutase (SOD), glutathione peroxidase (Gpx), and catalase [38,39,40]. Free Hb and heme in blood are bound by haptoglobin and hemopexin, and cleared by specific receptors in macrophages and liver [41]. Intracellular heme is bound and degraded by heme oxygenase (HO) [42].

### 3.2. Radiation and Oxidative Stress

Irradiation of biological tissues, such as in PRRT, leads to induction of oxidative stress. Thus, as described above, ROS and free radicals are generated by (1) direct interactions between the irradiation particles and molecules of the targeted biological tissue; and (2) secondary interactions between components released from the directly irradiated dead cells and surrounding cells and matrix molecules. The latter, secondary, response is also referred to as the “bystander effect”, *i.e.*, the irradiation causes damage to cells and tissues which are surrounding the targeted areas, but are not directly hit [43,44,45].

### 3.3. α_1_-Microglobulin (A1M)

#### 3.3.1. Structure, Expression, and Distribution

**Figure 2:**
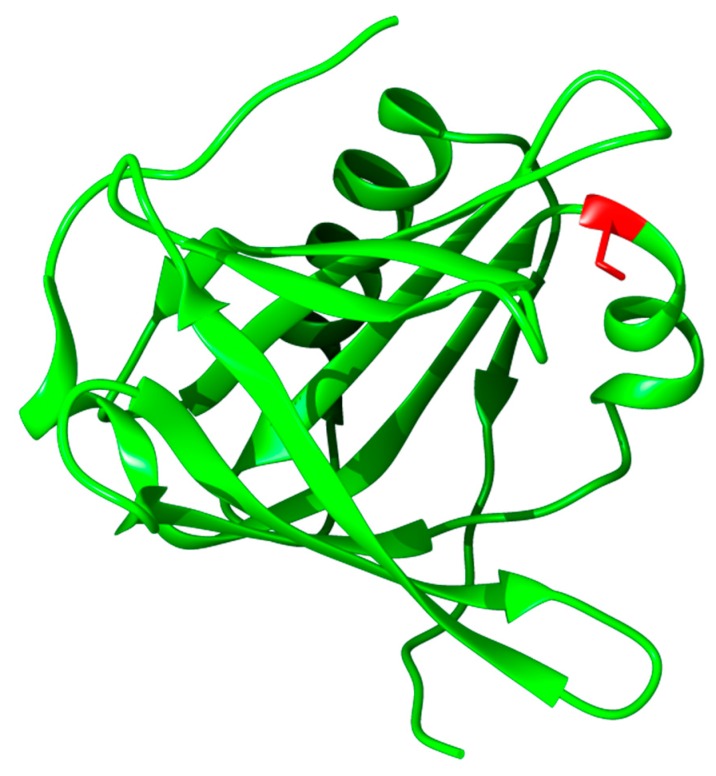
Three-dimensional rendering of A1M based on the published crystal structure [51]. The position of the C34 group (mutated to serine in the crystal structure) is highlighted in **red**. This is the critical site for the reductase, heme binding, and radical scavenging properties of A1M.

α_1_-Microglobulin (A1M) is a recently discovered physiological antioxidant, active both inside cells and in the extracellular compartments [46,47]. It is conserved in evolution and found in all vertebrates including birds and fish. A1M is a 26 kDa glycoprotein that belongs to Lipocalin protein family [48,49]. The Lipocalins are a group of >50 structurally related proteins in bacteria, plants, and animals, and are believed to originate from a common ancestor. They are one-domain proteins with a 150–190 amino acid-polypeptide and a common fold that consists of an eight-stranded β-barrel with a closed bottom and an open end at the top. The interior of the barrel usually serves as a binding site for mostly hydrophobic low molecular weight ligands [48]. The archetype lipocalin is retinol-binding protein (RBP) which transports vitamin A (retinol) in blood [50]. The structure of the major part of A1M was recently reported [51] and is shown in Figure 2.

The major site of synthesis of A1M is the liver [52], but all other investigated organs and cells express the protein although at a lower rate. The gene of A1M is denoted the α_1_-Microglobulin-Bikunin Precursor (*AMBP*) gene and encodes a continuous precursor protein consisting of A1M and bikunin, which are linked together during translation and in the ER lumen, but cleaved in the Golgi and secreted as two separate proteins with different functions [53,54]. Bikunin is a protease inhibitor and matrix component [55]. The co-synthesis is conserved in all cells and all species and the functional significance of the peculiar expression arrangement is yet unknown.

After secretion to the blood, A1M is rapidly extravasated and found in the extravascular compartment in most organs. Thus, a rapid equilibrium between intra- and extravascular compartments (T1/2 in blood = 2–3 min) is established [56] and a steady state concentration of 1–2 µM A1M in the plasma is achieved [57]. A1M is found both in a monomeric form and complex-bound to IgA, albumin, and prothrombin [58]. Being a small protein, A1M is equally quickly filtrated by the glomeruli of the kidneys and reabsorbed by the proximal tubule epithelial cells. Small amounts, however, escape this route and are excreted into the urine, amounting to approximately 5 mg/24 h [59].

#### 3.3.2. Biochemical Properties, Physiological Function, and Therapeutic Applications

A1M has been shown to have heme-binding, radical scavenging, and reductase properties [47] that suggest a physiological role as an antioxidant. Heme-groups can be bound by A1M [60,61] and proteolytic removal of a C-terminal tetrapeptide induces a pseudoenzymatic heme-degradation activity [60]. The free thiol group in amino acid position C34 (Figure 2) is the active site of a reductase/dehydrogenase activity of A1M [62]. Enzymatic reduction of free iron, heme proteins, and oxidized matrix and cytosolic proteins have been shown [62,63,64]. The reduction potential of the C34 thiol group also constitutes the structural and enzymatic basis of a radical binding mechanism that leads to covalent trapping of free radicals to lysyl and tyrosyl side-chains of A1M [65]. In line with this, human urine and amniotic A1M were shown to be yellow-brown due to covalent modifications [46,66,67].

As suggested by these biochemical properties of A1M, the protein has an antioxidant function in physiological situations with elevated oxidative stress. This has been verified in several reports that demonstrate that A1M can protect *in vitro* cell and organ cultures against oxidative damage from Hb, heme, and ROS [63,68,69]. Adding to the antioxidation potential *in vivo*, A1M was shown to accumulate in mitochondria via binding to mitochondrial Complex I stabilizing mitochondrial structure and maintaining ATP production during oxidative stress [70]. Also, upregulation of the *AMBP*-gene by elevated concentrations of ROS, hemoglobin and heme was reported *in vitro* and *in vivo*, suggesting an increased antioxidative protection potential during physiological oxidative stress [71,72].

A probable role as a radical- and hemescavenging antioxidant has led to the proposal of A1M as a therapeutic drug in diseases where elevated levels of Hb, heme and ROS constitute the major pathological insult [47]. Thus, infusion of A1M was shown to successfully treat placenta and kidney damage in the oxidative stress-related disease preeclampsia *in vivo* in sheep and rabbit models [73,74]. A1M could also inhibit glomerular damage induced by Hb-infusion in rats [75].

## 4. A1M in PRRT

### 4.1. A1M Protects against Radiation-Induced Tissue Damage

Two previous publications from our group demonstrate that A1M can protect bystander cells against irradiation-induced damage *in vitro* [64,76]. Using an experimental setup that allowed irradiation of a minor fraction of a human hepatoma monolayer, leaving the major part as bystander cells, it was shown that alpha-particle irradiation induced significant oxidative stress, cell death, apoptosis, and cell cycle arrest in the bystander cells. Addition of A1M to the culture medium reduced the cell death and inhibited apoptosis, oxidation biomarker formation, and upregulation of stress-response genes.

### 4.2. Infused A1M Is Localized to Kidneys in Vivo

To allow protection of radiation-exposed tissue-components in the kidneys during PRRT, a method to increase the concentration of A1M in kidney tissues should be developed. It was demonstrated several years ago that a large part (27%) of A1M was localized to the kidneys in rats *in vivo* 45 min. after intravenous (iv) infusion [56]. It was recently demonstrated in mice that exogenously infused A1M and the somatostatin-analogue octreotide, used clinically in PRRT, are co-localized in kidneys primarily in the proximal tubules, during the first hours after iv infusion [35]. Figure 2 illustrates the co-localization in the kidney cortex and on sub-cellular level in a proximal tubule after 20 min. In the study by Ahlstedt *et al.* [35] it was also shown that A1M appeared intact and full-length, suggesting that the protein still had functional activity.

### 4.3. Protection Hypothesis and Proof-of-Concept Experiments

The biological properties of A1M suggest that the protein may be employed as a co-treatment drug to protect against damaging side-effects on the kidneys during PRRT. Indeed, protection of kidney tissue from direct irradiation damage and/or bystander effects may allow a higher clinical dosage of the active peptides and hence a more efficient treatment of targeted tumors. This hypothesis is based on several sets of published results as described above. However, Proof-of-Concept experiments need to be performed to (1) ensure that kidney glomerular and tubular functions are significantly protected by A1M at various clinically relevant doses of radiopharmaceuticals while effects on tumors are maintained; (2) evaluate and optimize dosage regimen of both components. Such studies are presently undertaken at our laboratories.
